# Deep learning-based prediction of PFAS toxicity in zebrafish

**DOI:** 10.1038/s41598-026-54973-4

**Published:** 2026-06-06

**Authors:** Kanagaraj Ramasamy, Viswanathan Nallasamy

**Affiliations:** Department of Electronics and Communication Engineering, Mahendra Engineering College (Autonomous), Namakkal, India

**Keywords:** Soft error rate, Combinational circuit, Hypergraph partitioning, AND-INV graph, Single event transient, Engineering, Mathematics and computing

## Abstract

One such problem that is important in designing reliable circuits is soft due to high-energy particle strikes in combinational circuits. Soft errors affect the configuration bits that determine the circuit routing and logic and produce permanent errors. Though soft errors are temporarily natured, the constant repetitive soft errors of configuration or routing bits can cause permanent functional errors, unless addressed. To overcome this challenge, an extensive SER reduction mechanism is introduced in this study. The approach successfully combines Evolutionary-based Failure Probability analysis, logical implementation via AIG, and multi-level hypergraph-based partitioning. Circuit partitioning is performed by the METIS tool, and logical implementation is provided by the ABC tool. This is a continuous improvement of circuit designs to maximize the SER. These techniques are applied to the ISCAS’85 benchmark circuits, and the results demonstrate a significantly smaller average SER decrease of 23.7% compared to previously reported techniques. The proposed approach highlights its robustness in the design of complex architectures with several flexible examples of circuit designs. This is a practical and scalable method in modern integrated circuits to reduce SER and significantly enhance the reliability of the circuits against a soft error.

## Introduction

**Fig. 1:**
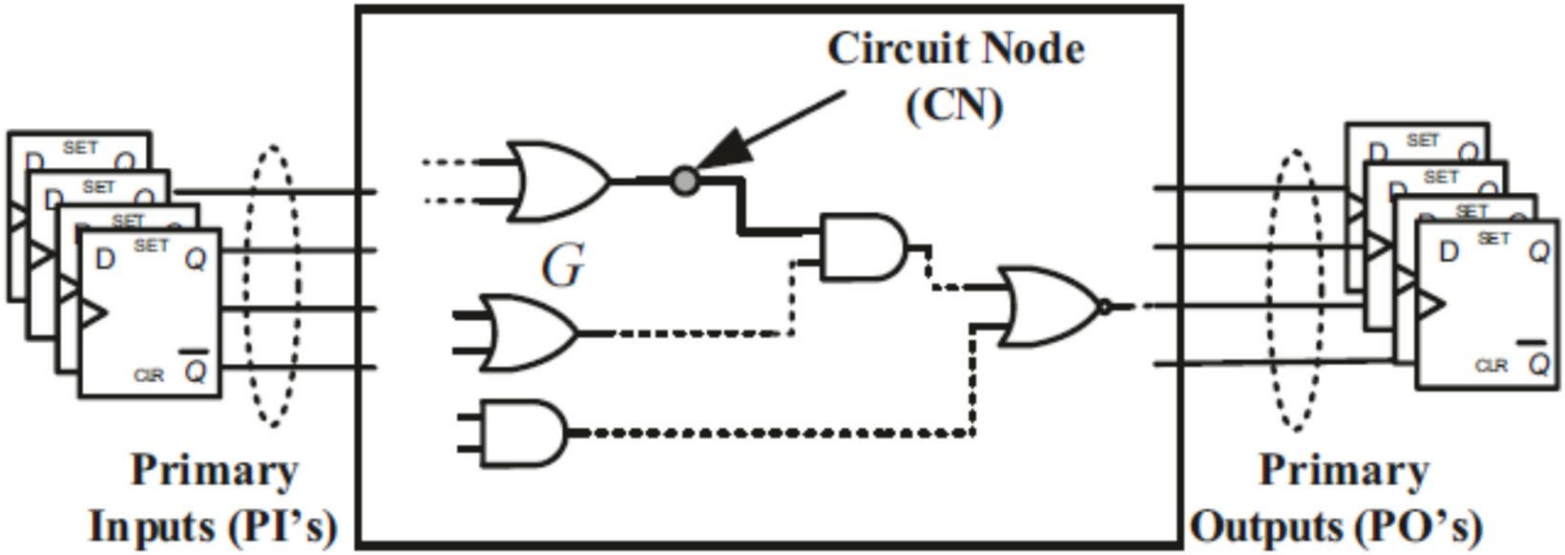
Combinational circuit.

Advanced technology of fabrication of digital circuits and transistor scaling down to the nanometer size have put the reliable operation of an integrated circuit at serious risk. Some of these enhancements stem from components such as reduced node capacitance, reduced power supply voltage, reduced threshold voltage of transistors, and higher operating frequency^1^. A transient voltage spike known as a Single Event Transient (SET) can result from a highly energetic particle striking a semiconductor device in a combinational gate^2^. The transient pulse might go to the storage parts through any variety of logic gates. One of the main factors that has the potential to impact combinational circuit reliability in recent years has been SET^3^. The (Fig. 1) represents the oriented general view of the main inputs (PI) and main outputs (PO) of a combinational circuit and gates representing the combinational logic.

A SET may be observed in the output of the inbuilt logic gates in addition to the relationship between PIs and internal gates. Each of these points within the circuit is referred to as a circuit node (CN). The set that contains every CN is known as the circuit node set (CNS). Each variable taken into consideration in POs may capture a SET generated at any circuit node within the circuit node set (CNS).

Because of the decrease in the significant charge and distance within junctions brought about by technology scaling, a high-energy particle strike that affects multiple adjacent cells causes Multiple Bit Upsets (MBUs) and Multiple Event Transients (METs) in the combinational circuitry elements and sequential elements, respectively^4^. After moving through the circuit’s gates, via the stroke gates to the flip-flops, transitory faults may cause an error. The term “soft error” refers to this type of error, while the term "soft error rate" (SER) describes the degree to which the circuit is susceptible to soft errors. Soft errors are temporary and do not produce physical harm, but permanent errors are caused by irreversible hardware failures like old age, defects in their manufacturing process, or constant radiation exposure. With repeated soft errors in configuration or routing bits, a long-term functional failure can be experienced unless fixed, yet the actual hardware is still intact. A soft error occurs in a circuit cell when a storage cell captures the generated transient pulse, resulting in an erroneous value. But during propagation, a few well-known masking effects may have an impact on several SET features^5^. The decreasing scale of technology nodes makes reduced critical charge and higher circuit density much more critical to the vulnerable range to soft errors, and SER mitigation is becoming a critical design consideration.

On the one hand, as stated in^6^, a circuit’s reliability (R) and failure probability (FP) are correlated as in Eq. (1).

1 *R=1-FP*

and yet another way, SER can be expressed as in Eq. (2).

2 $$\mathrm{S}\mathrm{E}\mathrm{R} = \mathrm{F}\mathrm{P} \times {\mathrm{R}}_{\mathrm{P}\mathrm{H}} \times {\mathrm{R}}_{\mathrm{e}\mathrm{f}\mathrm{f}} \times {\mathrm{A}}_{\mathrm{c}\mathrm{i}\mathrm{r}\mathrm{c}\mathrm{u}\mathrm{i}\mathrm{t}}$$

Where the entire silicon layer of the circuit is represented by A_circuit_, the particle’s impact rate per unit area is represented by R_PH_, and the percentage of particle impacts that cause a charge to be generated is represented by R_eff_^7^. As a result, Eq. (3) is an adverse relation between the circuit SER and reliability:

3 $$\mathrm{R} = 1-(\frac{\mathrm{S}\mathrm{E}\mathrm{R}}{{\mathrm{R}}_{\mathrm{P}\mathrm{H}}} \times {\mathrm{R}}_{\mathrm{e}\mathrm{f}\mathrm{f}} \times {\mathrm{A}}_{\mathrm{c}\mathrm{i}\mathrm{r}\mathrm{c}\mathrm{u}\mathrm{i}\mathrm{t}})$$

**Fig. 2:**
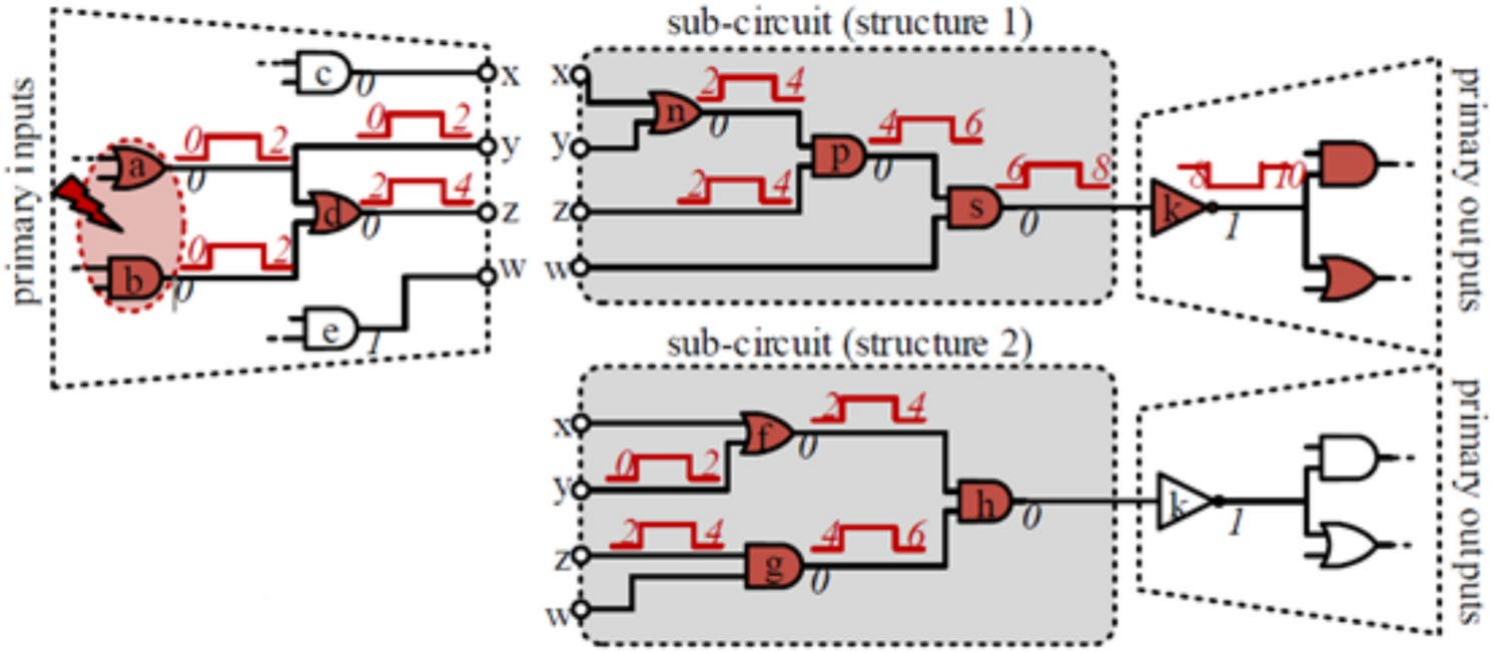
Sub-circuit architectures’ impact on the spread of transient faults.

Therefore, a reduction in the circuit SER is required to raise circuit dependability. In VLSI digital circuits, METs are predicted to occur more often than SETs in the modern era of technology scaling^8^. To design digital circuits with acceptable reliability criteria, one of the key problems is to introduce an effective method for reducing digital circuit SER when METs are present. An illustration of how different sub-circuit architectures affect the spread of transient faults is provided in (Fig. 2). In this instance, the MET propagates across two different sub-circuit structures (structures 1 and 2) after beginning beyond the sub-circuit at gates (a) as well as (b) between 0 and 1, having a width of 2ns. The transient faults propagate to structure 1’s output (with a width of 2 ns), while those of structure 2 are masked and do not propagate to that structure’s output. Therefore, using different sub-circuit architectures would disguise the transient faults that propagate across a sub-circuit.

To verify the effects of circuit restructuring on its dependability, the SER of the circuit must typically be recalculated (either a small part or the full of the circuit). However, under these circumstances, SER calculations for large circuits with METs present take a very long time. As technology becomes smaller, circuits become more prone to these kinds of errors, requiring efficient techniques for SER reduction. Achieving a balance between efficiency, area, and power limits while preserving circuit dependability is the fundamental problem facing SER reduction today. Due to shortcomings in failure probability analysis and circuit partitioning, traditional methods frequently fail to provide an ideal solution. The many interconnections and varied capabilities of contemporary combinational circuits necessitate the development of novel techniques to improve SER robustness.

This work introduces a multi-level hypergraph-based circuit partitioning mechanism as a novel solution to the problems with existing approaches.

When this technique is used, the main circuit can be broken into smaller more manageable parts. This is a contrast to the conventional approaches that cannot be used to calculate a Failure Probability (FP) for each Primary Output (PO) in the sub-circuits independently, and thus, the SER reduction plan is more focused and specific.

Also, there are a lot of logical constructions of sub-circuits that do the same job, and such constructions are investigated by the AND-INV Graph (AIG) method. This stage makes sure that the SER reduction procedure complies with space and circuit time requirements. Iteratively, improved evolutionary-based Failure Probability analysis is used to increase the SER reduction’s accuracy and efficiency. The identification and selection of the best circuit architectures is made easier by this iterative analysis, leading to a more reliable and customized SER reduction solution. This work actively tackles the issue of SER in combinational circuits by summarizing an in-depth methodology based on evolution-driven Failure Probability analysis, AIG-based logical implementations, and multi-level hypergraph-based circuit partitioning.

The evolutionary Failure Probability analysis, combined with AIG-based logical restructuring and multi-level hypergraph partitioning, presents a unique and complementary approach: mask, structure, and analyze with evolutionary techniques. Through collective use of these technologies, effective, scalable (reduction of SER) can take place.

The proposed framework of SER reduction comprises three major components: (i) multi-level partitioning using hypergraph partitioning, (ii) logical restructuring using AIGs, and (iii) Failure Probability analysis using evolutionary methods.

The following are the designed work’s primary contributions:

- To provide a multi-level hypergraph-based partitioning technique that divides intricate circuits into smaller, more manageable sub-circuits, hence increasing the effectiveness of SER reduction.
- To investigate several logical implementations using the AND-INV Graph (AIG) scheme, making sure that SER reduction complies with circuit timing and area limitations.
- The goal is to iteratively apply an enhanced evolutionary-based Failure Probability analysis that will greatly increase SER resilience by facilitating the selection of ideal circuit designs.
- To demonstrate the effectiveness of the suggested model in practical situations by validating its performance using ISCAS’85 benchmark circuits.

This research has a three-step workflow. To start with, multi-level hypergraph partitioning is used to decompose the combinational circuit. Second, every obtained sub-circuit is recreated with numerous logical realizations of the AIG. Lastly, an evolutionary analysis of failure probability is implemented in order to choose the sub-circuit implementation that reduces the total soft error rate.

## Related works

The drawbacks of traditional SER assessment of LSI networks are addressed by Song et al.^9^ in terms of a machine learning approach using a backpropagation neural network (BPNN). The suggested approach gives better results than the traditional evaluations by 7.5% and is well correlated with experimental results. The method significantly saves the calculation time and can be used to assess the SER performance of a circuit larger than 10,000 gates. It has many applications in radiation hardening and circuit design, and is a fast and efficient alternative to tedious simulations.

The impact of ageing, process variations, and bias temperature instability (BTI) for nanoscale CMOS circuits was investigated by Li et al.^10^ In the analysis of NanGate’s 45 nm process, they suggested an enhanced Soft Error Rate (SER) computation method, studied the threshold voltage drift and designed and simulated delay and critical charge for commonly used logic gates. From the results, it can be concluded that the process variables evaluated (when added to the BTI) had a significant effect on SER, whereas these effects for individual variables were not as significant. A novel calculation approach for SER showed the highest growth rate of 11.6%, demonstrating the importance of including synergistic effects when evaluating the dependability of nanoscale circuits. Tsoumanis et al.^11^ present a comprehensive study of SER from electrical characterisation of TCAD and an electrical mask study using SPICE. They explore the impact of interconnection delay and of static timing analysis (STA) on SER estimates and discuss the importance of accurate timing models.

The work investigates SER shift with device node scaling using benchmarks fabricated in 45 nm as well as 15 nm technologies.

By locating common minterms in Boolean algebra, Roy et al.^12^ suggest a unique soft error tolerance approach for combinational circuitry, which lowers failure rates. This approach covers these minterms with different cubes in order to maximize the chance of logical masking. The likelihood of a circuit failure has significantly decreased, according to the experimental data. The suggested method outperforms current soft error tolerance techniques by effectively returning the best answer among simplified sum-of-products using tabulation or K-map. Compared to traditional methods, it produces a 16% decrease in circuit failure and greatly speeds up the process of generating streamlined circuits that have lower failure rates.

In combinational logic, Hajisadeghi et al.^13^ tackled SETs that lead to METs, a problem made worse by scalability to ultra-low feature sizes. With a cross-layer strategy, their CLEAR method makes use of hardening techniques like resizing and fault masking in addition to an algorithm for optimal cell placement that utilizes local displacements and linear programming. According to experimental data, there is an average 42.9% decrease in double event transients (DETs), which might increase to 51.1% and 68.9% with additional hardening techniques. All of these reductions were made with very little overhead and no negative impact on the area.

To improve upon the Triple Modular Redundancy (TMR) and Genetic Solution (GS) techniques, Tan et al.^14^ provide a General Efficient Triple Modular Redundancy (GE-TMR) method for combinational circuits. A noteworthy SER reduction (>88%) is achieved by optimizing multi-objective functions using the suggested Solution Distribution Optimized NSGA-II (SDON) technique. A hybrid MIX approach that combines GE-TMR and GS also performs better than individual optimization, particularly in the region of 100–200% area overhead.

To identify vulnerable locations in a circuit, Georgakidis et al.^15^ proposed a Monte-Carlo-based approach for estimating SER, taking into account all possible masking strategies and utilizing layout information. We investigated two layout-aware strategies: Triple Modular Redundancy (TMR) based and All-to-All. The model based on triplets (TMR) for the finest delicate parts, compared to All-to-All, provided a better SER reduction. This method produced improved area and performance results in addition to increasing SER reduction. The study demonstrated the efficacy of TMR-based mitigation with a thorough SER analysis for combinational logic that took Single Event Multiple Transients (SEMTs) and layout details into account.

Recent SER mitigation techniques and physical SER reduction techniques include brain-inspired hybrid-grained scrapping mechanisms of SRAM-based FPGAs, and holistic SER reduction strategies to clock distribution networks, pay attention to layout-specific hardeners, and run-time error-recovery^16,17^. The given approach proves beneficial since it incorporates both logical reorganization and evolutionary maximization without having to copy the hardware or waste large areas of space.

The proposed approach, in contrast to current SER reduction methods, integrates circuit partitioning, logic re-synthesis, and evolutionary failure probability analysis into an integrated framework, by contrast with all currently available methods of SER reduction that address only layout hardening (or redundancy, or logical masking)^18–22^. This allows optimization of target SER at the sub-circuit level without having to re-compute the entire circuit, unlike the methods used before.

## Materials and methods

This sub-section describes the suggested algorithmic process, relying on the signal graph representations and hypergraph modeling. There is no material involved physically; the method is just based on some computational techniques of SER reduction.

**Fig. 3:**
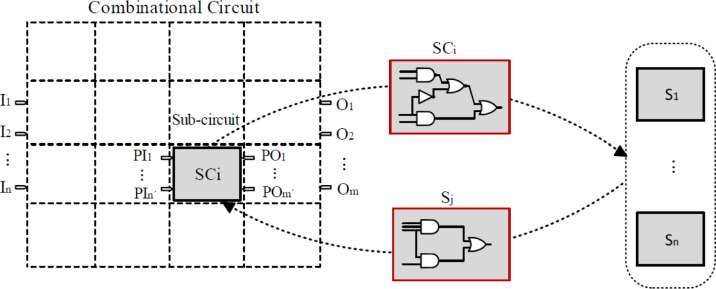
Framework of the proposed methodology.

The standard graph and hypergraph data structures are used in the proposed method to model the relationships of connectivity and partitioning in circuits. In this work, SER is calculated at the logical abstraction level and not directly calculated on the basis of physical layout parameters. Figure 3 provides an overview of the suggested technique for lowering the combinational circuit’s SER in the vicinity of METs. This framework illustrates how the suggested approach first splits the combinational circuitry into a few smaller sub-circuits (SCs). Next, different logic structures (i.e., n structures S_1_, S_2_,….S_n_ for SC_i_) are retrieved for each of the SC_i_.

### Circuit partitioning

Modern VLSI circuits are complex; one way to overcome this complexity in design optimizations is by partitioning the circuit into multiple components, each of which may be handled separately. In this work, the multi-level hypergraph-based partitioning approach is used. Compared to conventional graph-based techniques, this method more effectively represents the intricate interconnections within a circuit by utilizing hypergraph theory^23–26^. With the help of hypergraphs, relationships involving several nodes may be modeled, giving circuit dependencies a more realistic depiction. Upon partitioning the hypergraph, every sub-circuit is handled as a separate entity for subsequent analysis. The circuit’s manageability is improved by this division into simpler parts, which additionally facilitates specific measures for SER reduction more easily.

The initial stage in this technique is to model the circuit as a hypergraph, in which the connections between the logic elements (gates, flip-flops, etc.) are represented by hyperedges and the nodes represent the logic elements themselves. The mathematical definition of a hypergraph is as H = (V, E), where V represents the set of nodes and E indicates the set of hyperedges^27^. A subset of nodes v_i1_, v_i2_,..,i_im_ are connected by each hyperedge e_i_ *∈* E. Reflecting the connections within the circuit, the hyperedges represent the dependencies between nodes. Therefore, a circuit C with n gates is partitioned into k sub-circuits, and the subsequent scenarios need to be stated:

4 $$S{C}_{i} \in C\,\,\,\,\,\,\,\,\,\,\,\, i=\{\mathrm{1,2},\dots .,K\}$$

5 $$S{C}_{i} \cap S{C}_{j}\,\,\,\,\,\,\,\,\,\,\, i\ne j and i,j=\{\mathrm{1,2},\dots .,k\}$$

6 $$\sum_{i=1}^{k}S{C}_{i}=C$$

**Fig. 4:**
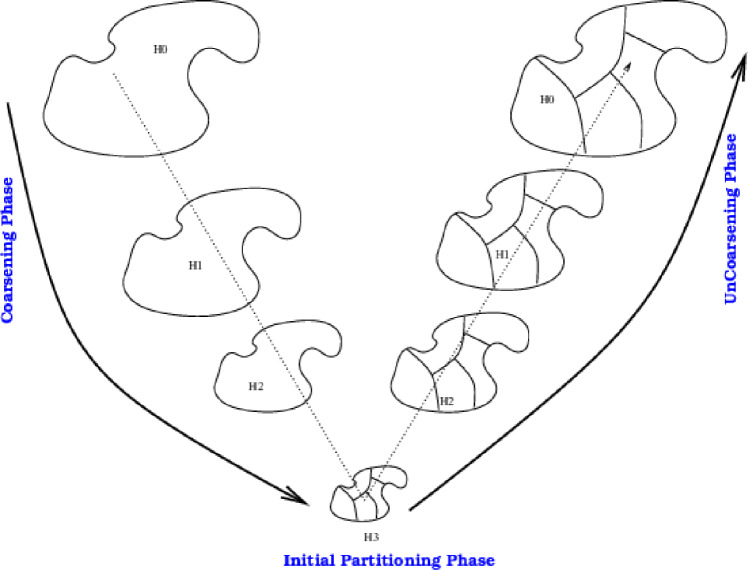
Phases of multi-level hypergraph-based circuit partitioning.

This study presents a multi-level hypergraph partitioning strategy that comprises of multiple unique stages, as depicted in (Fig. 4), each of which adds to the overall efficacy of the method^28^. The *coarsening* phase, in which the hypergraph is systematically reduced in size by node aggregation, is one important stage. This coarsening procedure is essential for controlling the intricacy of complex circuits so that effective partitioning may be achieved. The decreased set of nodes and hyperedges is represented by the terms V’ and E’, respectively, in the coarsening phase, which involves gradually coarsening the hypergraph H = V, E to create a coarser hypergraph H’ = V’, E’. In order to accomplish this reduction, nodes and hyperedges are combined while maintaining the crucial connectivity details of the original circuit. Heuristics are frequently used to direct the coarsening process in order to find nodes that can be combined without seriously impairing the integrity of the circuit depiction. Much like the original hypergraph, but on a smaller scale, the coarser hypergraph H’ keeps its structural features. This coarsening procedure efficiently reduces the complex circuit to a smaller, easier-to-manage version, which prepares the system for the next partitioning stages.

After coarsening, the coarser hypergraph is divided into an initial set of clusters during the *initial partitioning* step. A hypergraph partitioning technique designed specifically for the coarser form is used in this phase. The aim is to acquire a first partitioning of the coarser hypergraph, which functions as a foundation for additional refining in later stages. Refining the partitions thus obtained from the coarser hypergraph is achieved by running the uncoarsening phase. To restore finer-grained partitions for the original hypergraph, the coarser hypergraph that was first partitioned is progressively uncoarsed during this phase. In order to maintain the finer details of the circuit, uncoarsening entails mapping the partitions back to the initial set of nodes.

This is necessary to maintain the accuracy of the partitioning process at the original complex circuit, as the improved partitions will eventually be used on the original circuit. The multi-level hypergraph partitioning procedure is iterative, that is, it requires several repetitions of the coarsening, initial partitioning, and uncoarsening steps. An innovative partitioning algorithm is used to divide the hypergraph into more manageable, smaller sub-circuits. Well-known cost restrictions that can be utilized to divide the desired circuit include the number and dimension of each sub-circuit, and the cut-size. The goal of this approach is to provide a fair distribution of nodes among divisions while minimizing the cut size. Reducing the number of hyperedges that cross partitions, or the "cut size," is essential to preserving circuit functionality and cutting down on delays. Let P_1_, P_2_,…, P_k_ represent the partitions that resulted from using the algorithm.

An optimization problem is used to formulate the partitioning process:

7 $$Minimize Cut ({P}_{1},{P}_{2},\dots .,{P}_{k})$$

Assuming constraints that ensure an equitable allocation of nodes throughout divisions. Assume that x_ij_ is a binary variable that indicates if node v_j_ is a component of partition Pi. Cut size is defined as:

8 $$Cut \left({P}_{1},{P}_{2},\dots .,{P}_{k}\right)= \sum_{i=1}^{k}cut{(P}_{i})$$

9 $$Cut \left({P}_{1},{P}_{2},\dots .,{P}_{k}\right)= \sum_{i=1}^{k}\sum_{j=1}^{\left|V\right|}{({w}_{ij}x}_{ij})$$

Subject to the constraints:

10 $$\sum_{i=1}^{k}{x}_{ij}=1 \,\,\,\,\,\,\,\,\forall j\in V$$

11 $$\sum_{j=1}^{\left|V\right|}{({w}_{ij}x}_{ij})\le \frac{1}{k}\sum_{j=1}^{\left|V\right|}{w}_{ij}\,\,\,\,\,\,\,\,\ \forall j\in \{1, 2,\dots ..k\}$$

where the number of hyperedges that partition P has cut is indicated by cut(P_i_)*,* the weight *w*_*ij*_ indicates the significance of the link among nodes *v*_*i*_ and *v*_*j*_. In order to reduce the cut size, the partitioning method iteratively improves the node assignment to partitions. It is based on methods with recursive bisection and spectral partitioning, adjusting the partitioning assignment based on the eigenvectors of the hypergraph’s adjacency matrix. At each stage, the partitioning is made more refined, resulting in better quality and manageability of the sub-circuits. This iterative approach ensures that the resulting partitioning is effective and preserves the fine granularity of the original circuit while using a balance of coarse and fine granularity.

**Algorithm 1:**
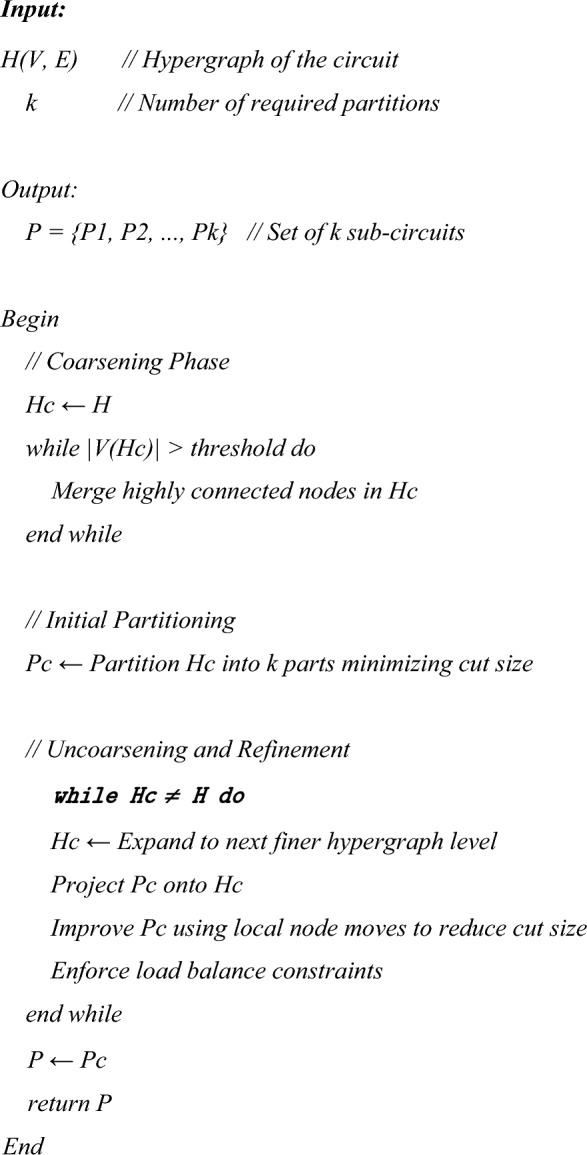
Multi-level hypergraph-based circuit partitioning.

**Fig. 5:**
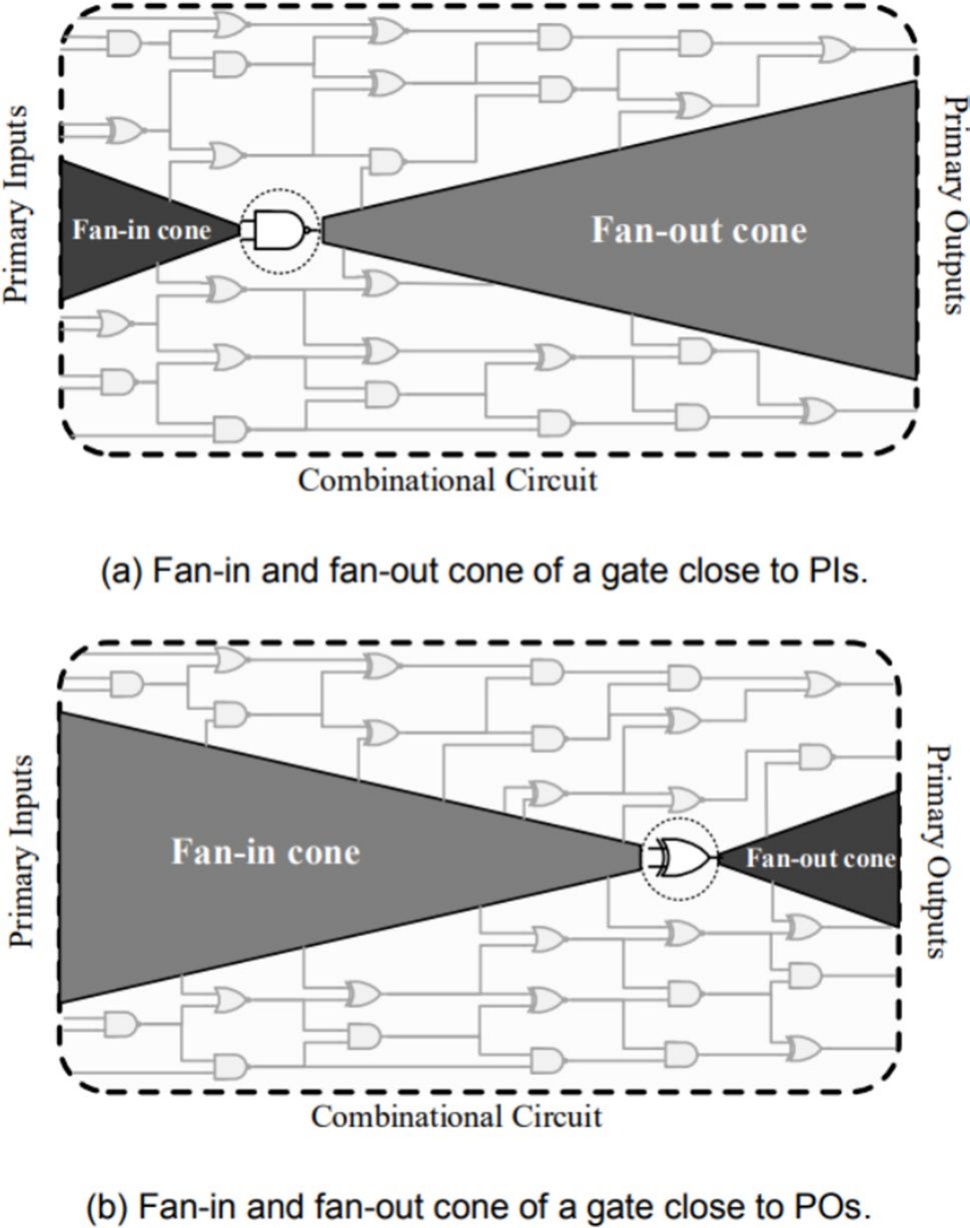
Combinational circuit with FIC and FOC.

The Fan-Out Cone (FOC) and Fan-In Cone (FIC), that is, the output and input relationships of sub-circuits respectively, are the basic concepts used in circuit partitioning as shown in (Fig. 5). Cones like these are essential for comprehending how transient faults spread and assessing how sub-circuits affect the total SER. Any input nodes and the pathways that connect them that either directly or indirectly affect the behavior of a sub-circuit SC_i_ are represented by the Fan-In Cone of that SC_i_.

Let V_in_(SC_i)_ be the set of SC_i_ input nodes, and E_in_(SC_i)_ be the set of hyperedges that connect these input nodes inside the subcircuit. FIC is denoted as in Eq. (12).

12 $$FIC\left(S{C}_{i}\right)=({V}_{in}\left(S{C}_{i}\right), {E}_{in}\left(S{C}_{i}\right))$$

Every output node and all of its associated paths that are impacted by the behavior of an SC_i_ are included in the FOC of the SC_i_. LetV_out_ (SC_i_) is the set of SC_i_ output nodes, and E_out_ (SC_i_) is the set of hyperedges that connect these output nodes within the subcircuit. FOC is denoted as in Eq. (13).

13 $$FOC\left(S{C}_{i}\right)=({V}_{out}\left(S{C}_{i}\right), {E}_{out}\left(S{C}_{i}\right))$$

Every gate that possesses at least a single path to a PO is included in the set, in order to divide the circuit according to the POs. After that, the remaining POs build all of the sub-circuits by repeating this method. The sub-circuits are extracted from the main circuit using the following procedures:

*Constructing the primary graph*: Every node (N) in the main circuit graph (G) represents a gate in the circuit, and every edge (E) depicts the link between two gates that are connected directly.

*Levelization of Circuit*: Using a modified linear topological sorting technique, each node’s level is determined. Every relation from vertex u to v in a conventional topological sorting algorithm places u before v; however, in the proposed ordering technique, vertex v comes before u in such a situation. Since the POs in the circuit are the first to be encountered by the sorting framework and constitute the main output list (POL), they are all in level (0).

*Tagging the nodes:* Every node (n) that is visited when traversing backwards from a PO to the PIs will receive a tag (T). In this stage, we give each PO a unique tag, and we keep doing so for every node along the path between PO and PI (s). Consequently, until the reverse traversal from a PO to PI(s) is completed, every node would be assigned a similar tag as the PO tag. When two nodes have a mutual tag, it indicates that the circuit has at least a single path connecting them. Therefore, Eq. (14) is used to produce a set of tags for each node:

14 $$TL\left(n\right)=T(n)$$

wherein T(n) is each traverse’s allocated node tag.

*Considering each PO into account when extracting the input cones*: Up to this point, every node has one or more tags that were acquired in the preceding stage. Finding the nodes that have a minimum of a single path to a given PO is necessary to acquire the input cones of each PO. As previously stated, the presence of a path between two nodes indicates that their tag lists contain a mutual tag. Thus, the input cone for the specified PO can be defined by comparing the PO tag with those in each node’s tag list. One among the POs from the POL is initially selected to carry out that task. Next, the tag list of the circuit’s nodes is compared with the PO tag that was obtained in the previous step. Any gate that has a resemblance among its PO tag and any tag in the node’s tag list (TL(n)) is regarded as a sub-circuit part.

*Constructing the sub-circuit*: At this stage, we possess the necessary data regarding the nodes situated in the POs’ input cone. To construct a sub-circuit, we must also be aware of the locations of each node that makes up an input cone. This is accomplished by combining the data from steps two and three. The following equation is used in the design of each sub-circuit that contains the PO node i (sub_i_).

15 $$\forall i\in POL \to Su{b}_{i}=P{O}_{i}$$

16 $$\{n(TL\left(n\right)=TL\left(T\left(n\right)\right)and \left(TL\left(n\right)=TL\left(P{O}_{j}\right)\right)\}$$

Wherein PO_j_ stands for the sub-circuit that connects PO (j) and PO (N).

The resulting sub-circuits possess the subsequent attributes:

(a) There is only one PO per sub-circuit.

(b) The quantity of POs in the main circuit and the quantity of sub-circuits are equal.

(c) Sub-circuits are totally independent; all that is required to determine the PO probability in a sub-circuit is its gates.

Calculating the probability for each PO individually can be done by using the Genetic Algorithm-based Failure Probability analysis. Multi-level hypergraph partitioning allows local SER optimization and removes complicated full-circuit recomputation since the circuit is broken up into small sub-circuits.

A combinational circuit is, for example, initially represented as a hypergraph, where the logic gates are the vertices, whereas the signal nets are the hyperedges. The splitting step associates closely related gates into sub-circuits with a minimal number of inter-partition connections.

### Probability analysis- genetic algorithm

**Fig. 6:**
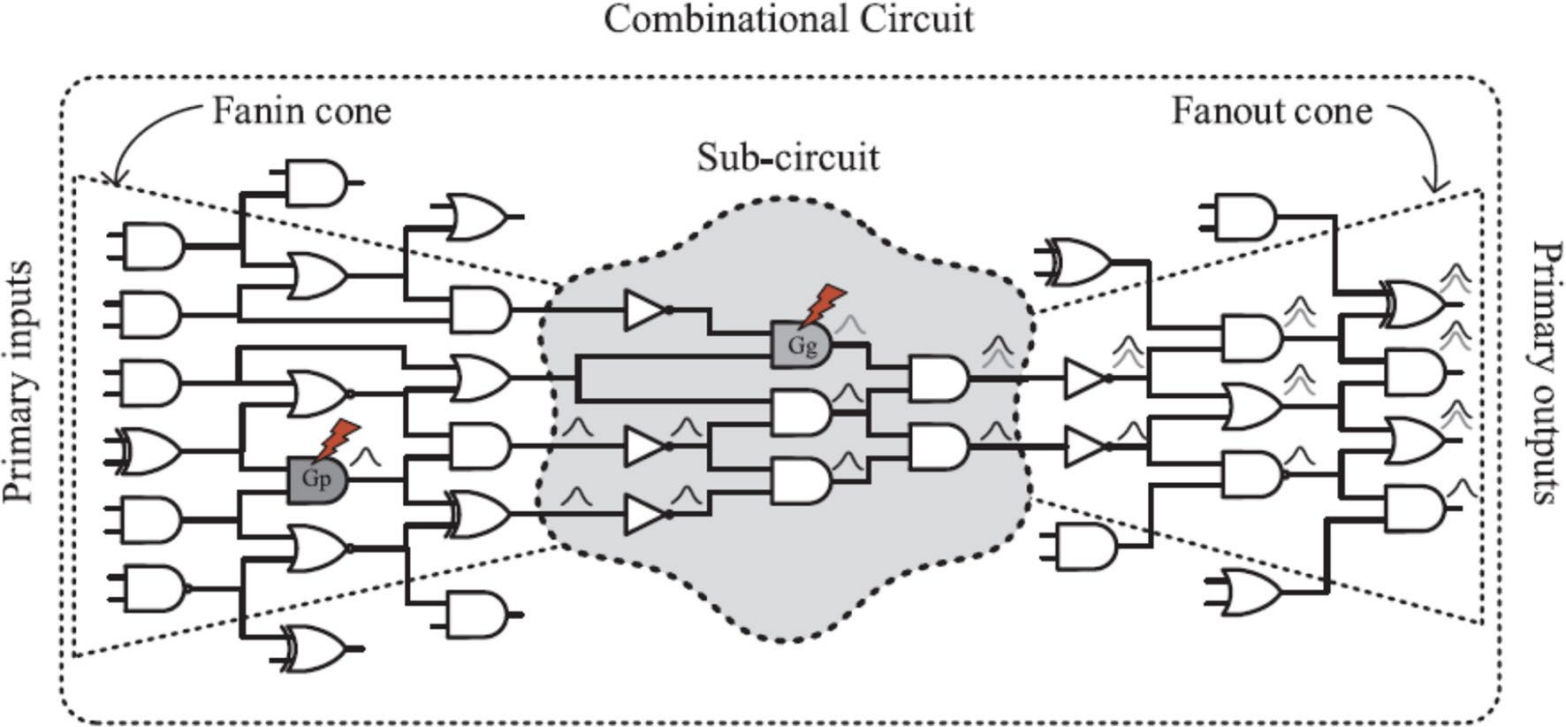
Illustration of transient fault generation within a sub-circuit and its propagation through the fan-in and fan-out cones toward the primary outputs.

The Failure Probability analysis is an iterative process using evolutionary-based approaches to assess alternative sub-circuit implementations and select a sub-circuit implementation that produces a minimum amount of transient faults and minimizes consumers of these faults, resulting in a reduction of the total SER. By considering the FICs and FOCs of the sub-circuits, the sub-circuits’ impacts on the soft errors are responsible for the generation as well as propagation of transient faults. The characteristics that the sub-circuits generated show that the METs originated at the sub-circuit’s gates, spread, and arrived at POs inside the FOCs of sub-circuits. The propagating portion of the sub-circuits shows that, in the case of each sub-circuit, the METs are generated at the FICs gates and then spread and arrive at POs in the sub-circuits and associated FOCs. Figure 6 demonstrates how the fault originates in the sub-circuit’s gate G_g_ and travels to the subcircuit outputs before reaching the POs. Additionally, (Fig. 6) illustrates the process by which a transient fault propagates throughout the sub-circuit. This diagram illustrates how the transient fault originates at gate G_p_ in the sub-circuit’s FIC and travels through the subcircuit to the POs. The transient faults generated in the fan-in cone can cause the propagation of the transient faults through the sub-circuit logic and reach the primary outputs, contributing to the overall SER, as illustrated in (Fig. 6). They propagate and reach POs via the sub-circuit and its FOC, respectively. The arrangement of the sub-circuits and where they are inside the main circuit determine these effects. The Genetic Algorithm-based Failure Probability analysis (GAFP) parameter has been implemented in order to assess the impact of the sub-circuit SC_i_ on the formation and propagation of faults in the primary circuit.

Eq. (17) is employed to estimate the probability that the sub-circuit (P_gen_) may generate a transient fault. Here, Yg is the likelihood that a transient fault produced at gate g will reach a significant fraction of the primary outputs. Transient fault generation in a sub-circuit within the circuit is calculated based on the above fault generation and propagation model as follows.

17 $${P}_{gen}=\sum_{i=1}^{N(PG)}LEG({G}_{i})$$

where N(PG) is the number of gates in the sub-circuit and LEG(G_i_) is the likelihood that a soft error would arise as a result of the generation of a transient fault in gate G_i_ of the sub-circuit that arrives at the POs. Eq. (18) describes the likelihood of the transient fault propagating.

18 $${P}_{prop}=\sum_{j=1}^{N(PI)}LEP(P{I}_{j})$$

Wherein LEP(PI_j_) is the likelihood of a transient fault occurring at the flip-flops at POs, fault propagating via the subcircuit, and fault generation in the FIC of the subcircuit input (PI_j_).

**Fig. 7:**
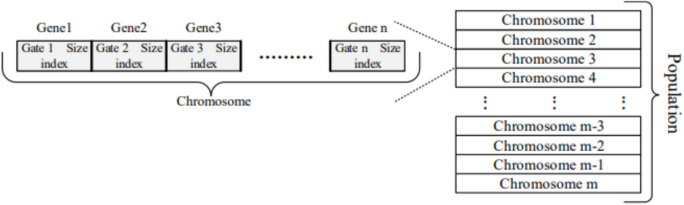
The GA’s population and encoding mechanism.

We employ a genetic algorithm-based optimization technique to lessen the SER of the sub-circuit. The genetic method often encodes the result into a binary code known as a chromosome^29^. Rather than starting with a single approach, the search starts with an initial population, which is a random group of chromosomes. A typical gate is thought to have k distinct sizes, which are represented by the numbers 0 to k-1 on the chromosome, correspondingly. Each gene is thought to represent the size of a single gate, as shown in (Fig. 7).

A fitness value is allocated to every chromosome, which is directly linked to the optimization problem’s objective function^30^. The genetic algorithm’s objective in the suggested approach is to reduce the sub-circuits’ SER. A chromosome’s goodness is demonstrated by its fitness. Each chromosome’s fitness correlates with the relevant path’s susceptibility, as per Eq. (10). The fitness function assesses a path’s susceptibility to soft errors. The higher the error-propagation rate, the higher the vulnerability. This function assesses the fitness of a path by treating it like a chromosome. The most vulnerable and rapidly propagating chromosomes will be chosen to create the next population (subset of pathways). Ultimately, a subset of pathways will be identified by the GA as the most vulnerable. The remaining paths are regarded as the ones that cause errors.

19 $$Fitness\left(chromosome\right)=Vu\mathrm{ln}erability(corresponding path)$$

To produce a new generation of chromosomes, the population is subjected to three operators: crossover, mutation, and selection. The population’s state gradually becomes closer to the state with globally optimal solutions after a sequence of iterative operator applications. One of the primary functions of the GA algorithm is selection. To create the next population, the most desirable chromosomes from the initial population are chosen using a selection operator. The selection operator searches for viable options to raise the population’s average quality. The ranked-based roulette wheel is employed by the suggested GA. The purpose is to increase the probability that the GA will identify the most susceptible paths. The selection probability of chromosome i, or p_i_ in this selection procedure, is defined as in Eq. (20). The fitness value of chromosome i is shown by the variable f_i_, and the population size, pop-size, is equal to 10. Premature convergence may result from the roulette-wheel selection with regard to the fitness values of the chromosomes in the population. Large disparities in fitness values increase the likelihood of reselection for the best-fitting chromosomes while decreasing the odds of selection for the other chromosomes. As a result, the GA’s ability to identify the most vulnerable pathways will function better.

20 $$S=\sum_{i=1}^{pop-size}{f}_{i}$$

21 $${p }_{i}=\frac{{f}_{i}}{S}$$

By swapping the genes of two parent chromosomes—which are chosen by the selection operator—crossover produces offspring. The genes of two parents are replicated to the initial portion of the offspring until the crossover point is chosen at random by this operator. Genes are then exchanged and copied into the last portion of the offspring after the crossover point of the two parents. Mutation causes individuals (chromosomes) in the population to randomly vary or change. It is a crucial operator that supports both the population’s continued variety and the exploration of new search space areas. Until a termination condition is met, this generational process is repeated. Three primary operators are applied to the population of solutions in case the termination criterion is not met, resulting in the creation of a new and potentially improved population. To show that one GA generation has been finished, the generation counter is incremented.

**Algorithm 2:**
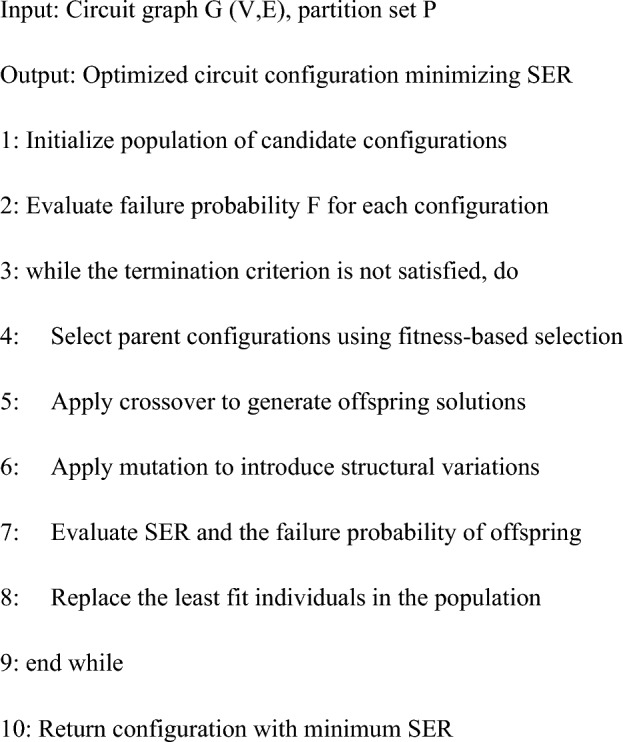
Evolutionary failure probability optimization.

### Logical sub-circuit designs

**Fig. 8:**
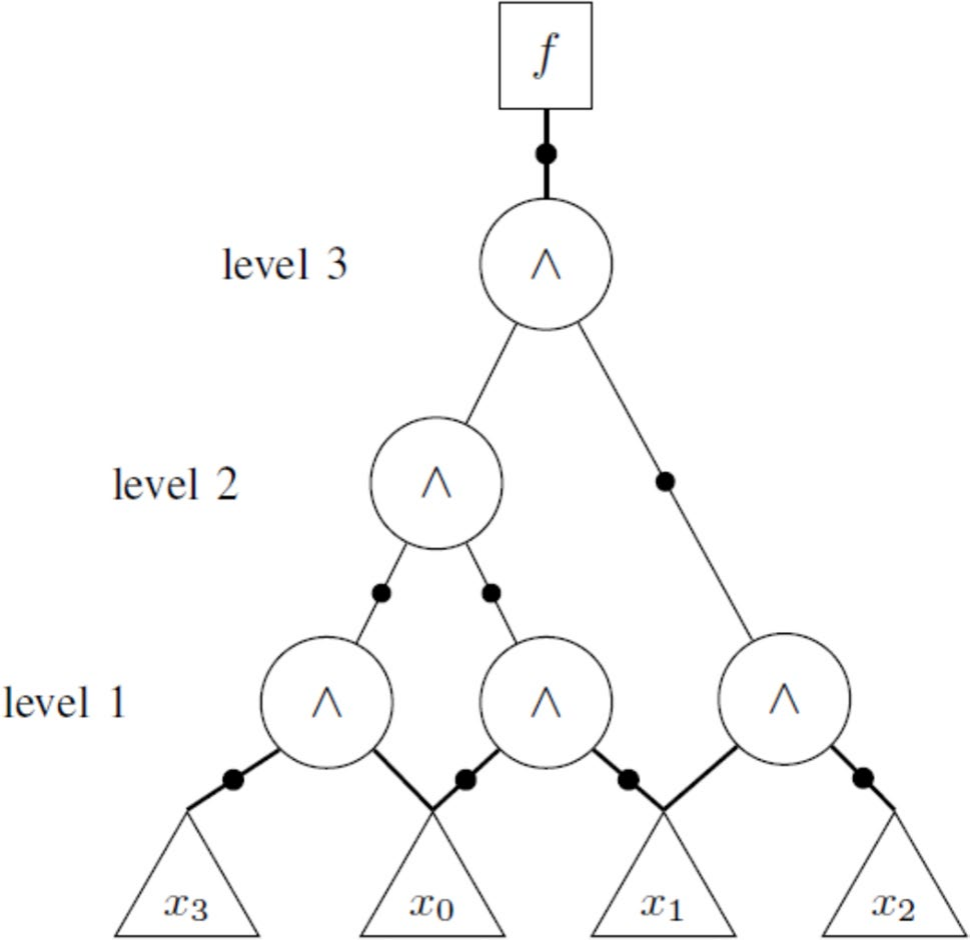
AND-Inverter graph for function f.

Following the successful partitioning of the hypergraph, each sub-circuit is examined separately. This will make the circuit more manageable and enable targeted interventions to reduce the SER. Various circuit implementations that have the same functionality are separated from each other. Implementations of sub-circuits exhibit dissimilar effects on the development and propagation of transient faults due to their varied logical architectures. Logical representations of the AND-INV Graph (AIG) approach^31^ are then examined within each sub-circuit to make sure that SER reduction complies with area and circuit timing constraints. A directed acyclic graph G = (V, E) with three different forms of nodes is called an AND-Inverter Graph (AIG), as shown in (Fig. 8).

The first form represents a distinct terminal node that acts as the principal output and has no outgoing edges. The second form, which represents primary inputs, lacks any incoming edges. A Boolean AND operation is represented by the third form, which has two entering edges and one exiting edge. A regular edge that represents the functionality as it is really implemented, and an additional edge that represents its negation, are the two types of outgoing edges that these AND nodes have. AIG is defined as:

“An AIG over the primaryinput variables X = {x_1_, x_2_,…, x_n_} and with theprimary output variables Y = {y_1_, y_2_,…, y_m_} is a directedacyclic graph G = (V(=V_X_ *∪* V_g_ *∪* V_Y_})E *”.*

As a result, the AIG technique might be used to gather many representations of a specific logical operation while considering the desired design factors. Different implementations of a given sub-circuit SC are extracted in this study by first creating an AIG for the subcircuit function, after which different implementations are extracted based on design specifications and data from the default cell library. Ultimately, a collection of these implementations is referred to as API_SC_. The AIG scheme permits the exploration of a wide variety of structurally different, but functionally equivalent implementations to choose designs with better fault masking properties.

## Results and discussions

### Simulation Setup

The system specifications included utilizing a machine with an Intel Core i7 processor, 16GB RAM, and an NVIDIA GeForce GTX 1080Ti GPU. Python is used to accomplish the suggested method. The ISCAS’85 benchmark circuits were employed to test the approach. In addition, the 45-nm Nangate Open Cell Library^32^ is employed. Every SER measurement was conducted under the assumption of a 56.5 m^2^s^−1^ sea level neutron flux^33^.In the evaluations, the circuit is divided into a number of tiny sub-circuits using the METIS tool described in^34^. The ABC tool^35^ is used to extract different logical representations of sub-circuits having a comparable logical function. Using the ABC tool, various sub-circuit implementations are retrieved in the tests. This study uses a logic synthesizing script described in^35^ to retrieve various representations of a sub-circuit with a similar logical purpose. These scripts carry out several rewrite, refactor, and balance command iterations. Multi-level hypergraph circuit partitioning using METIS to minimize the cut size and sub-circuit balancing, whereas the ABC tool is used to create multiple AIG-based logical implementations based on repeated logic rewriting and optimization. METIS v5.1.0 was configured with the default recursive bisection parameters and a target partition being the number of primary outputs. The version used was ABC v1.01 with ten iterations of the rewrite, refactor, and balance program on each sub-circuit.

The genetic algorithm enhances optimization accuracy at a minimum computational burden since it tries to refine sub-circuits by continuing with the iterative process. Through a SER evaluation run on the local circuit (as opposed to the actual circuit) in sub-circuits, and not on the actual circuit, the runtime is linear to the size of the circuit, thus making the method practical to very large designs.

### Evaluation of the proposed approach

C432 is common in SER studies because the middle level of the logic depth of a large logic defines its good choice to analyse the effect of masking faults when it has reconvergent fan-out paths. By contrast, C880 is very rich in logic levels and more reconvergent nodes and thus represents larger designs in industry that have large combinational designs. The ISCAS’85 benchmark suite’s circuits C432 and C1355 have been chosen in an effort to reduce SER. These circuits were selected with particular considerations that support the goals of the study. The C432 circuit, despite its smaller size, exemplifies a compact digital circuit with its 36 primary inputs, 7 primary outputs, and 160 gates. This decision makes it possible to analyze the suggested SER reduction technique in detail on a circuit with a moderate level of complexity. However, C880 is a more complex and demanding testbed because of its bigger circuit size, which has 653 gates, 33 major outputs, and 41 primary inputs.

The ISCAS85 benchmark circuits have been chosen because they have been widely used in reliability tests, and because they are structurally diverse, thus permitting a consistent and significant comparison of SER reduction methods.

**Fig. 9:**
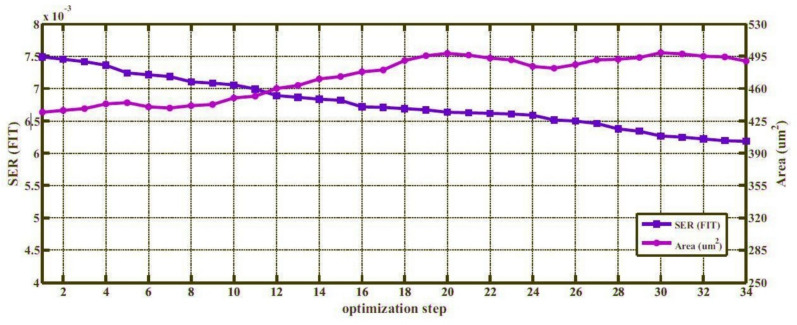
Effects of the suggested approach on the C432 benchmark circuit’s SER and Area.

**Fig. 10:**
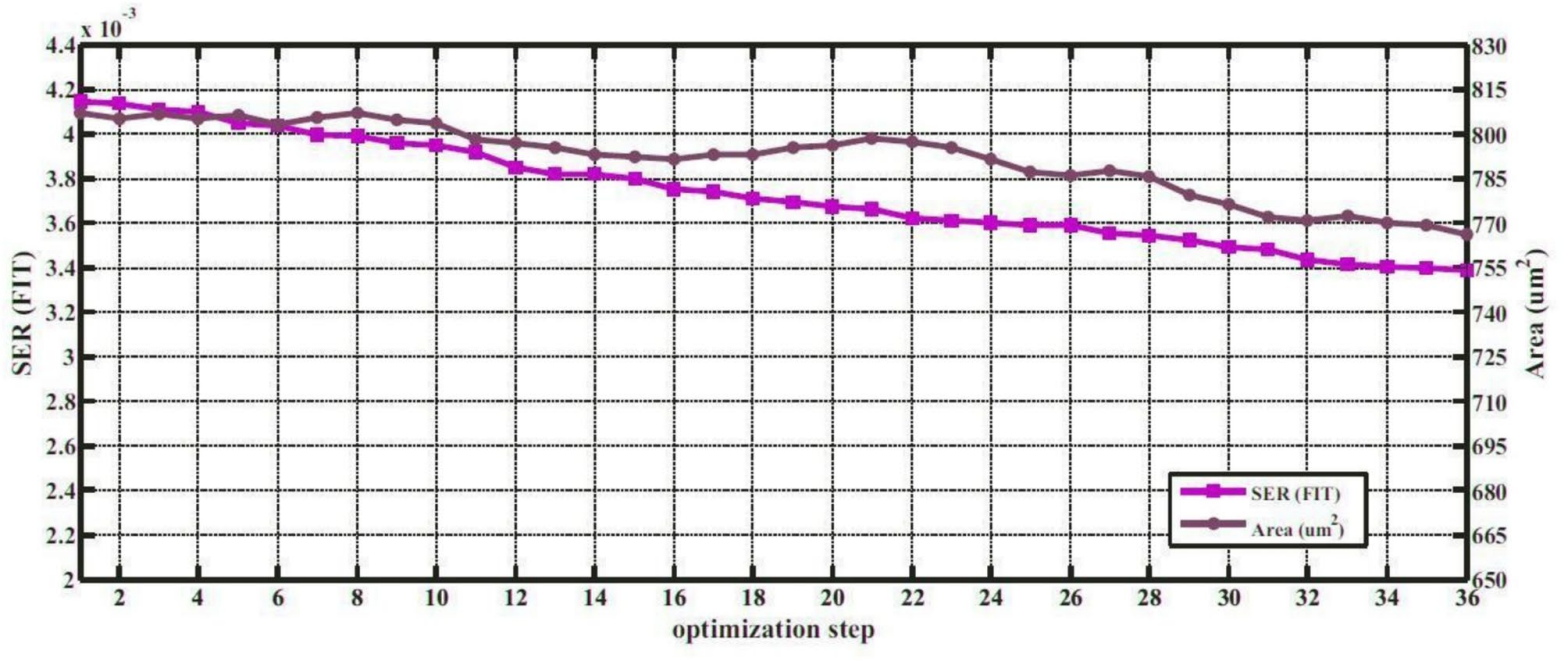
Effects of the suggested approach on the C880 benchmark circuit’s SER and Area.

The SER and Area of the C432 and C880 benchmarks, which are determined at every stage of the optimization procedure using the suggested scheme, are displayed in (Fig. 9 and 10), respectively. These figures demonstrate that, for the most part, SER is decreasing. Nevertheless, in certain instances, SER is decreased more quickly. Steps 4, 11, and 31 in the C1355 benchmark and steps 4, 15, and 27 in the C432 benchmark, for instance, both show a sharp decline in SER. Every circuit’s SER reduction is contingent upon its circuit structure as well as the arrangement, dimensions, and placement of the sub-circuits that are removed at each stage. The findings demonstrate that the suggested approach works well in the majority of the enhancement process without requiring a complete recalculation of the circuit SER.

**Fig. 11:**
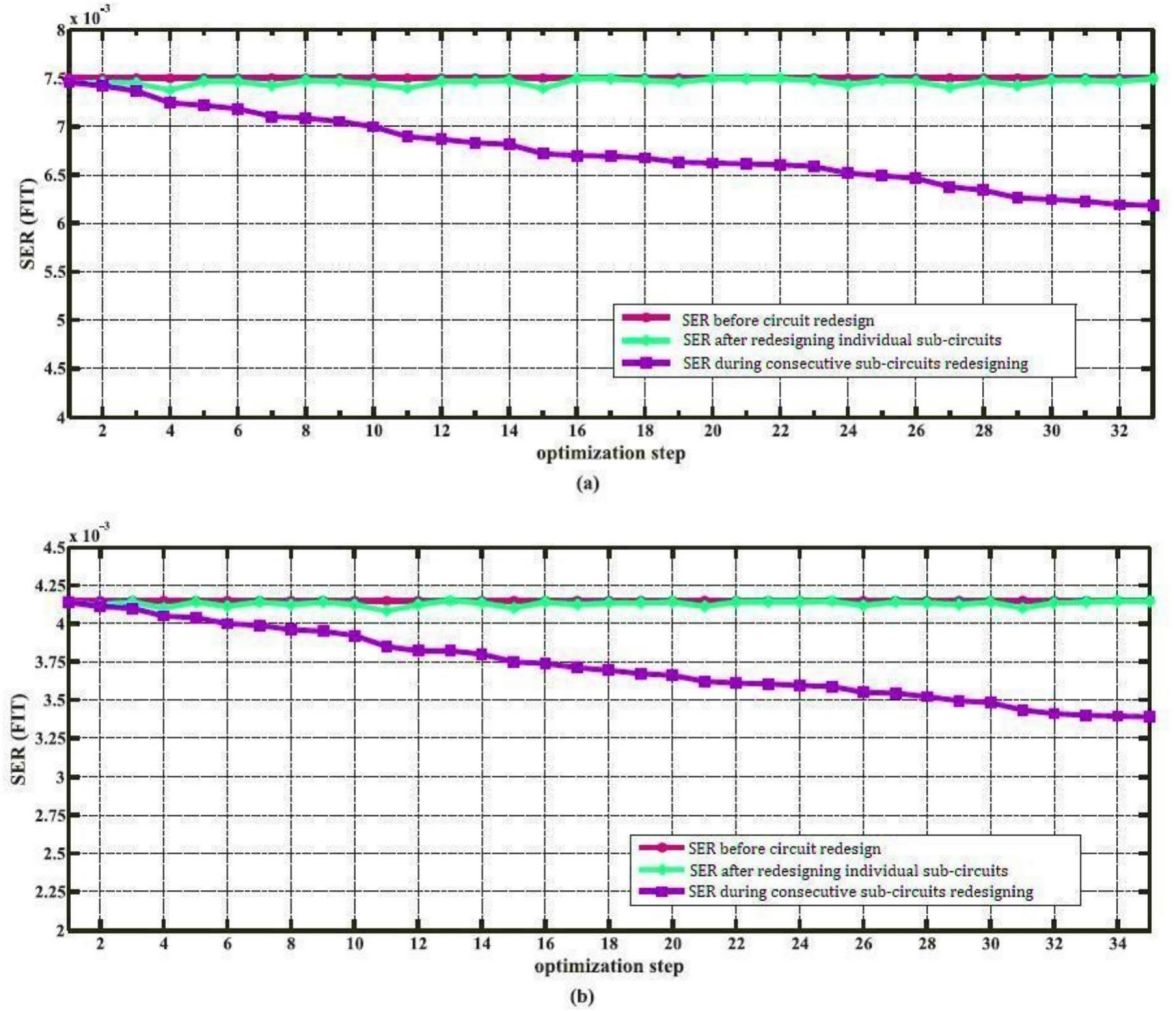
Sub-circuit redesigning’s impact on the circuit SER.

The soft error rate (SER) of the C432 and C1355 benchmark circuits is evaluated before and after individual sub-circuit redesign, as well as during sequential sub-circuit optimization, correspondingly, are compared in (Figs. 11a and b) varying sub-circuits have varying SER reduction values, which vary depending on how they are structured and where in the circuit they are located. As the sub-circuits are reorganized one after the other, the circuit’s SER is continuously decreased. Redesigning every sub-circuit considerably lowers the total circuit SER, as the result demonstrates.

The outcomes of the suggested technique on ISCAS’85 benchmark circuits are displayed in (Table 1). The suggested approach uses subcircuit redesign in an attempt to increase logical masking and decrease the likelihood that a transient fault will propagate through the circuit gates to the primary outputs of the circuit. Additionally, by sub-circuit reconstruction, the suggested model seeks to reduce the anticipated pulse width that reaches the circuit output primarily. As a result, there is a drop in the circuit SER and latching probability. The table indicates that the benchmark circuits’ average SER is decreased by 23.7%, with average area overhead and delay overhead being 5.83% and 4.94%, respectively. The average SER reduction of 23.7 percent with the proposed method is competitive with current methods and has much lower area and delay overhead relative to redundancy-based methods.

**Table 1:** Simulation outcomes using the ISCAS’85 benchmark circuits.

Bench	#PI	#PO	#Gate	#SC	SER reduction (%)	Area change (%)	Delay change (%)
C432	36	7	160	8	2.15E+01	−2.10E+00	3.21E+00
C499	41	32	650	33	2.40E+01	1.45E+01	1.20E+01
C880	60	26	512	26	2.65E+01	−2.86E+00	−2.80E+00
C1355	41	33	653	33	2.92E+01	2.32E+01	8.78E+00
C1908	33	25	699	35	2.10E+01	−3.92E+00	−1.79E+00
C2670	157	64	756	38	2.55E+01	−7.38E+00	6.02E+00
C3540	50	22	1467	74	2.85E+01	1.10E+01	7.91E+00
C5315	178	123	2115	106	1.95E+01	1.85E+01	6.77E+00
C6288	32	32	4507	226	2.42E+01	2.15E+01	1.88E+01
C7552	207	108	2534	127	2.75E+01	1.57E+01	5.43E+00
Average	–	–	–	–	2.37E+01	5.83E+00	4.94E+00

The SER measurements were performed several times so as to eliminate the changes brought about by the stochastic fault propagation in the genetic algorithm. The average change in SER per repetition was 23.7, and the standard deviation was 1.2 between the benchmark circuits, which showed uncertain results in the proposed method. The statistical analysis shows that there is low variability, and it proves the strength of the approach.

**Fig. 12:**
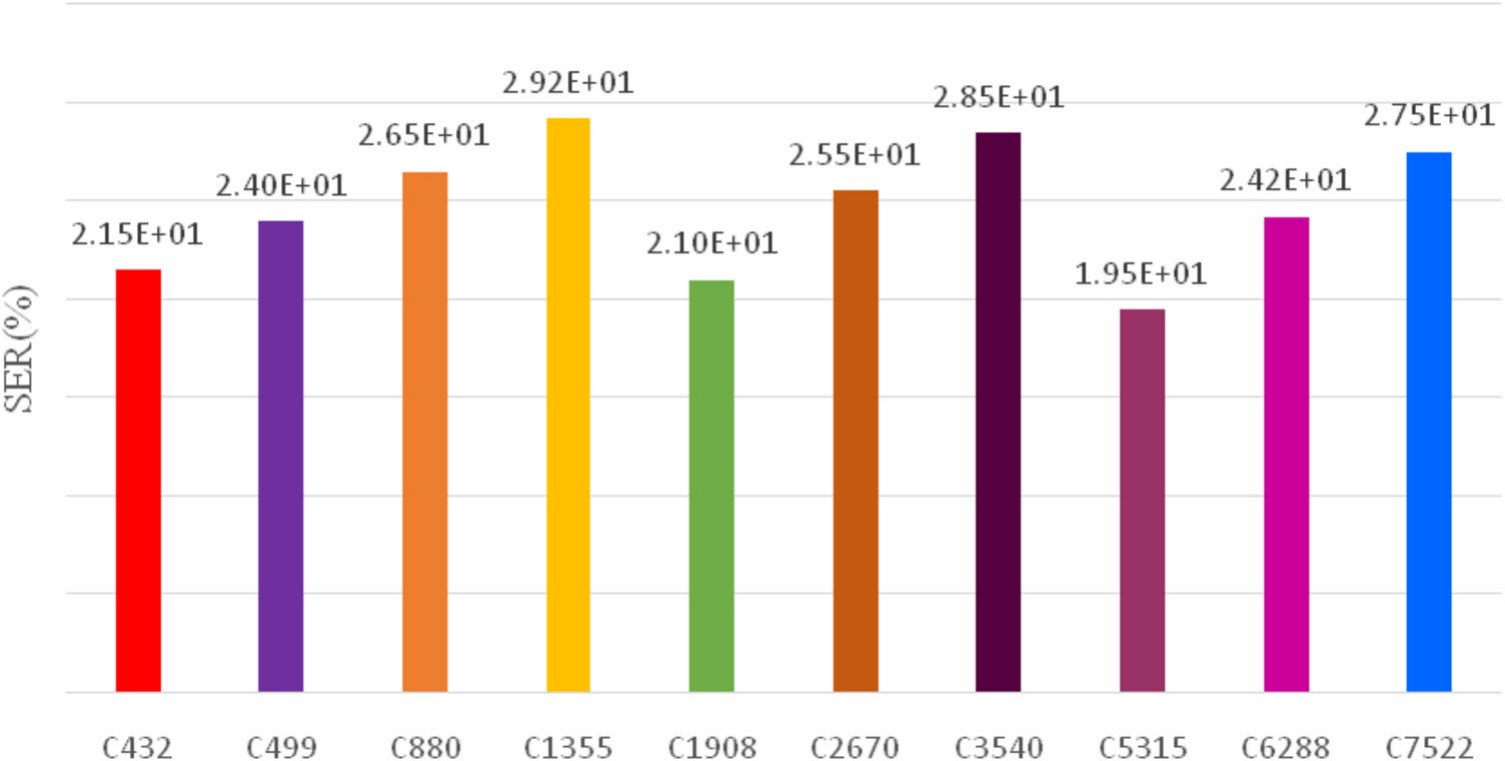
SER reduction.

The SER reduction percentages for the several benchmark circuits are displayed in (Fig. 12). Every data point on the graph illustrates the different levels of improvement by reflecting the precise SER reduction attained for a certain circuit.

The variation in SER between the assembly of various benchmarks indicates the flexibility of the proposed methodology to various circuit topologies and structural complexities. The observed differences between various circuits highlight how flexible the suggested methodology is to various circuit complexities and topologies. The percentages for SER reduction are higher in circuits like C1355 and C3540, indicating the technique has the potential to cope with complex designs. Using the suggested evolutionary-based Failure Probability analysis, the overall average SER reduction of 23.7% shows that the circuits’ reliability is significantly improved when applied to combinational circuits (Table 2). The finding suggests that the methodology offers enhanced resistance to soft errors in different benchmark circuits by optimizing the circuit designs.

**Table 2:** SER reduction on ISPD benchmark circuits.

Circuit	SER before (partitioning stage)	SER after logic restructuring and evolutionary analysis	SER reduction (%)	Area overhead (%)	Delay overhead (%)
b1243	1.24 × 10⁻^3^	9.42 × 10⁻^4^	24.2	5.5	4.8
b1788	2.87 × 10⁻^3^	2.14 × 10⁻^3^	25.4	5.9	4.9
b2013	3.65 × 10⁻^3^	2.78 × 10⁻^3^	23.8	6.1	5.1
Average	—	—	24.5	5.8	4.9

Although the primary assessment employs ISCAS’85 benchmarks for consistency with previous SER investigations, (Table 3) presents initial results of SER reduction obtained on specific ISPD benchmark circuits. The findings indicate that the suggested approach is not only suitable for the initial benchmark libraries but also offers the same level of SER reduction and low overheads.

**Table 3:** Comparison of the proposed method with existing SER reduction techniques.

Method / Reference	Partitioning-based optimization	Logic restructuring	Evolutionary / Optimization-based analysis	Main contribution / Reported outcome
Song et al.^9^ (ML-based SER estimation)				SER prediction framework; ~7.5% estimation improvement, no direct SER reduction
Li et al.^10^ (BTI and process-variation-aware SER analysis)				SER sensitivity analysis with up to 11.6% variation impact
Tsoumanis et al.^11^ (Layout-aware SER estimation)				Improved SER estimation accuracy
Roy et al.^12^ (Boolean restructuring for logical masking)				Approximately 16% reduction in failure probability
Hajisadeghi et al.^13^ (CLEAR cross-layer hardening)		(cell-level hardening)		42.9–68.9% reduction in DET
Tan et al.^14^ (GE-TMR with NSGA-II)		(redundancy-based)		More than 88% SER reduction with significant area overhead
Georgakidis et al.^15^ (Layout-based TMR mitigation)				SER reduction achieved with area/performance trade-offs
Proposed Method	Multi-level hypergraph partitioning	AIG-based sub-circuit redesign	Genetic algorithm-based failure analysis	23.7% average SER reduction with low area and delay overheads

The suggested approach will be compared with some of the representative existing approaches in (Table 2). In terms of techniques that achieve higher SER reduction, our procedure provides an optimum combination of multi-level partitioning, logical restructuring, and evolutionary optimization techniques, and achieves an average SER reduction of 23.7%, while also resulting in a low area and delay costs.

Careful production may be required using general synthesis and analysis tools with VLSI in industrial VLSI flows. Issues such as how to script the iterative AIG optimization as part of the ABC flow, how to manage memory and run time usage of large-scale circuits, and how to meet timing and area requirements become challenges. The suggested partitioning plan alleviates these problems by localizing the optimization to sub-circuits to enable its effective introduction into the existing design flows.

The iterative optimization process ends if either of the following two criteria is met: (1) if the difference between the SER reduction of a successive pair of generations is smaller than a specific value, ε = 0.5%, or (2) if the total number of iterations (generations, 50) is reached. This guarantees convergence, without incurring too much computational overhead.

The multi-level hypergraph partitioning process is composed of three steps: coarsening, initial partitioning, and iterative refinement. The overall time complexity is about O(|E| log|V|), where |V| is the number of nodes and |E| is the number of hyperedges. The partitioning stage was implemented in practice with the METIS library that offers efficient heuristic optimization of large-scale graphs. Based on empirical assessment, the total simulation time is less than 6% due to the overhead of partitioning.

The proposed framework considers spatial correlation among the neighboring nodes in the circuit graph to model Multiple Bit Upsets (MBUs) and Multiple Event Transients (METs). A transient fault may occur at several nodes in a single physical region when a high-energy particle strike occurs in that region. The failure probability model thus considers the joint failure probability of multiple node failures in the same partition region. This gives a more realistic representation of radiation-induced soft error and allows correlated effects of fault propagation to be captured by the framework.

The ISCAS’85 benchmark suite is commonly used to test the general performance of fault tolerance techniques, and future research will include more extensive testing of larger benchmark suites like ISCAS’89 and ITC’99 for further validation of scalability for current VLSI-based systems.

## Conclusion

The proposed method improves the reliability and fault tolerance of the circuit to soft errors and can significantly reduce the propagation time of short transient errors to primary outputs. A comprehensive and new approach for minimizing the SER in ICs is suggested in the proposed study. It addresses soft error problems from a holistic point of view, by integrating the multi-level hypergraph-based partitioning approach, the logical implementation by using the AND-INV Graph (AIG) scheme, and the Failure Probability analysis approach based on evolution. These approaches applied to ISCAS’85 benchmark circuits give excellent results (a mean SER reduction of 23.7%) when compared to existing approaches. Safety-related applications, such as aerospace systems, medical apparatus, and military systems, in which safe work under radiation is essential, are particularly appreciated for the suggested SER mitigation approach. The proposed method is demonstrated to be effective and also flexible across various circuit topologies, indicating the capability to withstand complex designs. The study provides a realistic and scalable approach to the complexity of contemporary integrated circuits, greatly enhancing circuit dependability against soft errors. A new benchmark for SER reduction techniques is established through evolutionary-based analysis, logical implementation, and partitioning, which opens the path for greater circuit resilience and dependability from soft errors. The current assessment will be based on ISCAS standards of 85 benchmarks to remain consistent with the previous SER research, but will be expanded to include libraries, based on ISPD benchmarks, in the near future. The following future extensions to this work include applying the methodology to sequential circuits, applying the methodology to SER estimation in the presence of layout, and co-optimization of power, performance, and reliability.

## Data Availability

The datasets used and/or analysed during the current study available from the corresponding author on reasonable request.
